# Material philology and Syriac excerpting practices: A computational-quantitative study of the digitized catalog of the Syriac manuscripts in the British Library

**DOI:** 10.1371/journal.pone.0320265

**Published:** 2025-03-31

**Authors:** Noam Maeir

**Affiliations:** The Digital Humanities Center, The Hebrew University of Jerusalem, Jerusalem, Israel; Israel Antiquities Authority, ISRAEL

## Abstract

This study explores the literary practice of excerpting in Syriac manuscripts through a computational-quantitative analysis, contributing to the emerging field of Syriac material philology. The primary objective is to offer a “big picture” charting of Syriac excerpting as a non-authorial literary practice. Using digitized data from the British Library’s Syriac manuscript collection, the study analyzes nearly 20,000 excerpts, introducing the Excerpts Per Manuscript (EPM) metric to quantify and compare excerpting practices across manuscripts. The results reveal that most manuscripts contain fewer than 20 excerpts, but a small number show much higher levels of excerpting, highlighting the immense intellectual and literary activities implicated in their production. These high-EPM manuscripts appear across multiple genres, indicating that excerpting was a widespread and essential cultural activity rather than confined to specific literary types.

The study also finds that manuscripts with the highest EPM values are concentrated between the 6^th^ and 9^th^ centuries CE, corresponding with a period of intense literary compilation in late antiquity. This pattern reflects the importance of excerpting in knowledge organization, aligning with broader trends in the canonization of texts within Christian, Jewish, and Greco-Roman traditions. The research emphasizes the limitations of earlier cataloging approaches, which obscure non-authorial practices by focusing on authors and texts. By reorienting data through computational analysis, the study provides new insights into the role of excerpting in Syriac manuscript culture. This approach demonstrates the value of digital tools in material philology, uncovering patterns that bridge genres and timeframes, and identifying high-EPM manuscripts as key sites of intellectual and cultural activity in the Syriac literary tradition.

## Introduction

Over the recent decades, philological studies of pre-modern books, or manuscripts, have been undergoing a shift in their understanding of the pre-modern manuscript, their cultural object of study. Traditionally, pre-modern manuscripts (mss.) were considered by philologists as vessels that carry literary materials composed by earlier authors. The recent shift, often associated with the rise of New or Material Philology, places its emphasis on the vessels themselves, considering them as proper literary and cultural objects [1,2]. Rather than just a carrier, material philology treats the manuscript more like a literary arrangement, composed of literary materials that are purposefully cut, arranged and decorated, within the general layout of a mosaic, which, in its totality, is considered a composition in its own right [3–7].

From the perspective of material philology, the study of manuscripts is not limited to the traditional study of authored textual materials, but it also involves the study of other kinds of literary practices that are involved in the production of the manuscript, and which will be termed here “non-authorial literary practices”. Non-authorial literary practices are literary practices, i.e., literature-making practices, that are not performed by “authors”, but rather by other agents, such as scribes [5], editors [8], compilers [9,10], readers [11,12], owners [13,14], etc. These practices include a variety of textual interventions within the main body of the manuscript, such as, excerpting [10,15–17], rubrications [8,17,18], re-attributions [4,19,20], erasures [21], etc., as well as in various marginal notes [4,22,23] and other “paratextual” interventions [24,25].

To illustrate this further, the production of a pre-modern manuscript, especially when it contains diverse literary material, can be likened to that of a YouTube compilation video, such as “top 90s hit songs”, “greatest soccer goals”, “best action scenes”, “funniest jokes by comedians”, “life changing motivational speeches”, etc. Each compilation is the production of a video compiler that selected and ordered individual clips found in videos across the internet, i.e., the non-authorial practice of video “excerpting”. The compiled video is often accompanied with background music that is not part of the original clips, along with images and texts that are inserted throughout the progression of the video. Aside from the compiled video and the various interventions performed within it, one also finds interventions outside and around the video, including a description section; a rich comment section; measures of the views, likes and dislikes of the video; various links to other websites; advertisements; suggested videos, etc. As cultural objects, the compilation videos can be studied in an attempt to reconstruct the original and fuller videos that were used by the compilers. At the same time, compilations can be studied in themselves as witnesses of communities of producers-compilers, (re)users, viewers, commentators, etc., and the cultural attitudes reflected in their individual practices. Similarly, the literary collections can be studied to reconstruct the original texts of the original authors – a classical philology approach – or to explore the literary performances of non-authorial agents and the cultural attitudes reflected in their individual practices – a material philology approach.

The study of non-authorial literary practices within the framework of material philology is still in its early stages. Scholars are calling to further investigate these practices by “working on the fine details” as well as “the big picture” [9]. Responding to this call, this paper will perform a computational-quantitative analysis of a corpus of Syriac mss. from the collection of the British Library (BL), in an attempt to contribute to the “big picture” charting of material-philology-oriented Syriac studies, and to the study of the literary practice of excerpting.

### Syriac manuscripts

Syriac Christianity is a complex religious-cultural-social system that spanned geographically across the world, with Syriac communities and their cultural objects and literary traditions going back at least to the 4^th^ century CE up until current times [26–30]. Various cultural artifacts bear the traces of a rich Syriac literary culture, as found, for example, in inscriptions, mosaics, incantation bowls, etc., and above all, in a vast corpus of Syriac manuscripts. Syriac manuscripts are found in the thousands across the world, recently estimated at more than 20,000 worldwide, and can be securely dated from the early 5^th^ century all the way until modern times [27]. The rich geographical and historical contexts in which Syriac Christians lived, offer a well-documented opportunity to study pre-modern manuscript and literary cultures.

The material philology orientation in the study of Syriac mss. is expressed most prominently in studies of a specific type of manuscript called the “multiple-text-manuscript” (MTM), a manuscript that is essentially a literary collection [3,9,31]. MTMs are very common in Syriac literary culture, they were produced throughout the 1,500 years of Syriac monasticism, and are often entitled with indicative Syriac terminology such as “*kūnnuŝā*” (ܟܘܢܫܐ) [3,10]. The literary contents of MTMs are wide ranging, and include biblical, theological-monastic, historiographic, hagiographic, and liturgical materials [3,16,17,19,20,25,32–35]. Interestingly, a large portion of the literature that is preserved in the collections is not original Syriac writings, but rather Syriac translations of original Greek (mostly Christian) texts, which serve as important, and sometimes the only, attestations of the original Greek [3]. Furthermore, MTMs exhibit a massive recycling of literary material, seen in the reuse of specific textual extracts, and even of entire collections [9]. A given Syriac literary compilation will present some measure of “dependence” upon other MTMs, reaching up to four or five degrees of dependency between compilations (i.e., a collection that incorporates material from another collection, which in turn draws from a third, and so on). Scholars argue that MTMs, and even sub-collections within them, can be considered as original compositions, as actual authored texts, with original arguments, narratives, epistemologies, and cultural attitudes [16,17,32,35]. Moreover, the classification of Syriac MTMs developed by recent scholars includes monastic, spiritual, exegetical, dogmatic, heresiological, historiographical, and biblical collections (see above). Nevertheless, as the study of MTMs and the literary practices interwoven throughout them is relatively young, the classifications, methods, results and their presentations are subject to ongoing refinement and adaptation.

This paper will take one non-authorial practice involved in the making of MTMs, namely, excerpting, and offer a “big picture” charting of its behavior, in an attempt to contribute to the growing study of Syriac MTMs and non-authorial practices.

### Syriac excerpting

Excerpting, the collecting and arranging of excerpts (Greek - ἐκλογή, pl. ἐκλογαὶ; Latin - excerptus, pl. excerpta), is a literary phenomenon that scholars have found to be particularly characteristic of the Christian literatures of late antiquity. Studies have recently placed Christian excerpting practices in relation to literatures of antiquity, both within a Greco-Roman cultural framework, as well as within the cultural patterns of the Abrahamic religions of the long late antiquity (until the 10^th^ century CE).

The act of collecting excerpts was a widespread literary practice, mentioned and performed in writings of the classical Greek, Hellenistic and Roman periods. During this timeframe, excerpting became increasingly associated with pedagogic and scholarly works, yet it is the Christian usages that brought excerpting into the front stage of their literary performances, as a proper practice of fundamental composition of literature – especially of polemical, educational, exegetical, and historiographical works [36,37]. The extension of the literary function of excerpting by Christians was understood by some as reflecting the decline of Hellenism and the spirit of free thought together with the rise of Christianity and its authority and canon-oriented religious worldview. Morlet argues for a more nuanced framework, in which a combination of factors – a biblical and Jewish textual tradition, a Hellenistic literary background, and a social context of contemporary religious controversies revolving around paganism and heterodoxy –together brought about the novel Christian compositional usage of excerpting [36].

Aside from contextualizing late antique Christian excerpting within Greco-Roman culture, it was also analyzed within the later historical timeframe of the long late antiquity, until the 10^th^ century CE [9,38], situated within the cultural space of the Abrahamic religions [39]. In this context, collecting excerpts was understood as participating in a broader cultural pattern of the Abrahamic religions, the “canonization of textual authorities” [9]. Syriac excerpting can be seen as a specific form of canonization of textual authorities, though, not in the negative sense, but rather as an original act of interpretation and knowledge organization, shared across the Abrahamic religions of the first millennium CE [9,38]. In sum, Syriac excerpting was situated within the Greco-Roman context, as well as the Abrahamic religions, and serving in various literary functions, including pedagogic, knowledge organizing, and proper authorial composition.

While the content and function of excerpts may vary widely, the basic performance of a literary selection is inherent in each excerpt. Indeed, the Greek and Latin terms used for “excerpt” (Greek - ἐκλογή; Latin - excerptus) are also employed in the meaning of “choice” or “selection”, so that a collection of excerpts can be understood simply as a collection of selections, textual selections [9]. While an excerpt would usually be considered as an extract taken from another text, for this study I will define an excerpt simply as a textual selection, regardless of its content – another authored text, a biblical book, an abridgement of a treatise, etc. In many ways, this definition of the excerpt is very close to the concept of a “textual unit” employed in quantitative codicology [40–42].

We have now singled out excerpting as the literary practice at the heart of this paper. Since this is a quantitative-oriented humanities study, a further zoom-in is required, in order to clearly define the meaningful quantitative unit, or feature, that will be used in the “big picture” charting. The process of defining the quantitative unit of analysis, i.e., “feature selection”, is heavily dependent on the measure of quantification of a field of study – i.e., use of quantitative methods, discursive norms for employing quantitative arguments, and the production of analyzable data.

### The quantitative method: Distant reading

The mass digitization of cultural objects conducted over recent decades inevitably impacts the data accessible for scholars of the humanities, and a new scientific horizon has emerged, offering not only access to never-seen-before amounts of cultural artifacts, but also new methods and perspectives by which one can analyze and represent traditional data [43].

Franco Moretti, a scholar of world literature and one of the first to fully engage in the quantitative study of literary objects, coined the term for a new humanities-oriented quantitative analysis as “distant reading” [44]. In its current usages, “distant reading” has become an umbrella term for various humanities-oriented, machine-assisted (computational) and quantitative analyses of texts, as opposed to “close reading” [45–47]. In Moretti’s words:

*One last thing that became clear* […] *was the enormous difference between the archive of the Great Unread, and the world of the canon. You enter the archive, and the usual coordinates disappear; all you can see are swarms of hybrids and oddities, for which the categories of literary taxonomy offer very little help. It’s fascinating, to feel so lost in a universe one didn’t even know existed* … [44]

As opposed to the canon-oriented close reading, Moretti describes his distant reading of the “archive of the Great Unread” (i.e., a massive literary database), as an experience in which the usual “categories of literary taxonomy” lose their usefulness. When entering the “Great Unread”, a few works that are considered the literary canon, or any clearly defined periodization, do not necessarily aid in making sense of the “swarms of hybrids and oddities”. In other words, the taxonomical loss, the blurring of the literary categories, that can be experienced via distant reading offers the opportunity to study cultural objects without being necessarily confined to a specific classification or categorization [48]. As such, distant reading presents material philologists the opportunity to study Syriac manuscripts without being limited to a classical philology (author-oriented) classification or a specific periodization. As the periodization of late antiquity is still a contested issue in current scholarship [9,38,49–52], distant reading can be useful for studies of Syriac manuscripts during this period.

Lastly, this paper performs a distant reading, in an attempt to demonstrate that DH practices can aid in further establishing emerging fields of study, such as that of non-authorial literary practices, which are especially concerned with re-describing their data according to new paradigms, not beholden to previous classifications.

### The discursive quantification of Syriac excerpting

As mentioned, the application of a humanities-oriented quantitative analysis within a specific field of study, is potentially beneficial when discursive norms in the field include the usage of quantitative arguments. The process of discursive quantification within the framework of material philology has already begun, marked by the usage of numerical data and arguments, even within Syriac studies [24]. Specifically addressing the analysis of excerpts, numerical arguments are not yet employed, though a discernable level of quantification is found. This is manifested in studies employing structural and quantitative descriptions of excerpts and titles [10,17], as well as the identification of excerpting patterns [32], presented as integral parts of their analysis. And so, the distant reading of this paper is not performed in a vacuum, but rather within an emerging quantitative framework. Still, as the quantification of Syriac excerpting is still in its early stages, the arguments must be treated cautiously and tentatively, assuming that their merit lies in the contribution to a cyclical process between close studies and distant readings concerned with exploring the uncharted territories of non-authorial literary practices.

Furthermore, as far as the production of analyzable data – another important aspect of the quantification of a field – databases that facilitate studies oriented towards material philology have already become available online [e.g., 53–55]. These databases are dynamic, constantly expanding to include thousands of manuscripts, and presenting an invaluable resource for researchers. However, it is crucial to note that the metadata within these databases remains inconsistent, making large-scale quantitative analyses of Syriac excerpting not sufficiently reliable at this point. Consequently, data about excerpting must be procured from other sources, the well-established catalogs and databases rooted in classical philology, which have been produced for more than 150 years. These catalogs offer a more reliable source for obtaining systematic and comprehensive information about Syriac manuscripts and excerpting specifically.

## Data: The catalog of Syriac Mss. in the British library and its digitization

One of the largest physical collections of Syriac manuscripts is found in the British Library (BL), with 998 datable mss., spanning from the 5^th^-19^th^ centuries CE [56]. The mss. of the BL collection are described and made accessible thanks to the detailed catalog that was prepared by William Wright in the later part of the 19^th^ century [57–59]. Remarkably, the systematic and detailed description provided by the catalog has yet to be matched, so that scholars of BL Syriac mss. consult the catalog regularly until this very day. From our 21^st^ century perspective, especially when considering non-authorial literary practices, Wright’s catalog has significant limitations.

David Michelson studied the “assumptions, discourses, and theology employed by Wright” in the preparation of the BL catalog:

Rhetorically, they [i.e., Wright and his predecessor, Cureton] represented their cataloguing and arranging of the collection as simply sorting through texts that had been “mixed up…by time and chance”.

…They were primarily interested in how materials preserved in Syriac might grant access to lost cultural patrimony of European Christianity, namely biblical or Greek texts.[60]

**Fig 1:**
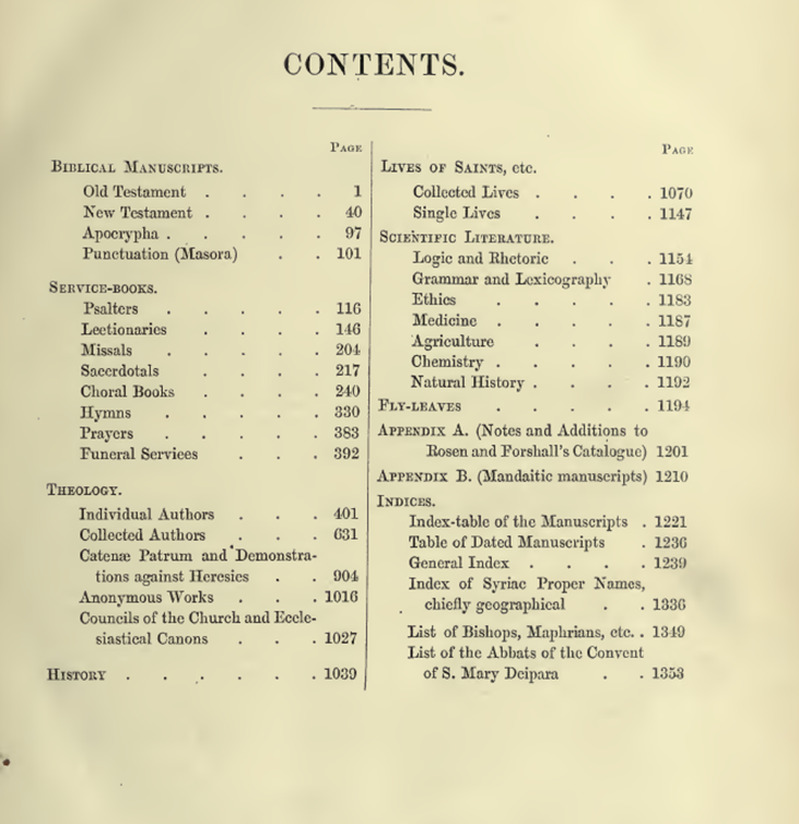
Wright vol. III, Table of Contents of Wright’s BL catalog.

**Fig 2:**
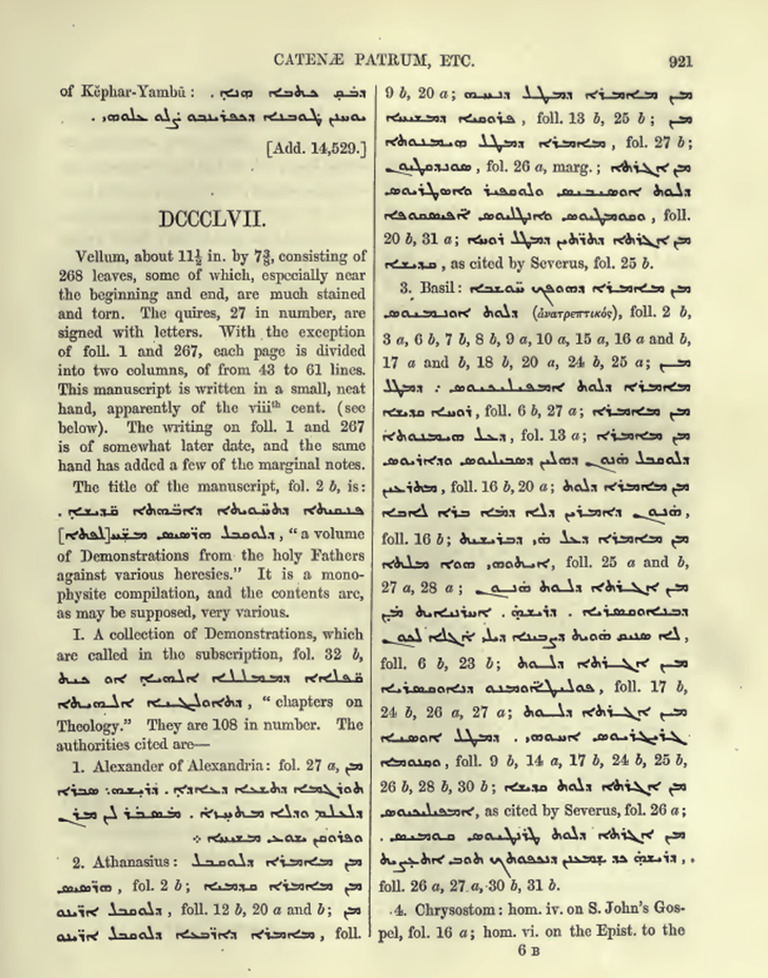
Wright, vol. II, p. 921. The first page of Wright’s description of Add. MS 12,155.

The BL catalog is governed by an author-oriented classification, according to which Syriac collections of (authored-)texts are treated as accidental mixtures that need to be transcended in order to bring into view the original texts, and in the process the original layout of the collection is lost. To illustrate, here are Wright’s table of contents and a sample of a description of a manuscript:

As seen in Fig 1, Wright grouped the BL mss. according to six general genres, which, aside from “History”, are each accompanied by several sub-genres. Let’s look at a sample description of a manuscript from the sub-genre “Catenae Patrum and Demonstrations against Heresies”, found within the general genre of “Theology”:

As seen in Fig 2, following a brief description of the codicological features of the manuscript (material, size, quires, script, dating, etc.), Wright gives the title of the manuscript, and characterizes it as a “compilation” of “very various” contents, and then turns to describe its contents. Wright divided the contents of the manuscript into 50 (L) sections – the image above portraying the description of the first (I) section, a literary collection which is entitled “chapters on theology” and includes 108 chapters. The contents of the 108 chapters are described by organizing the excerpts, 186 in total, according to the alphabetical order of their authors, and their texts, so that in the process, the original layout of the mss. is obscured. This is seen already in the first authority cited, a text by a “Alexander of Alexandria” (4^th^ century CE), which is found not at the beginning of the manuscript but on folio 27a. Thus, returning to the analogy with a YouTube video compilation, Wright’s cataloging logic would treat a compiled video (the literary collection) as a list of clips (excerpts), organized according to the alphabetical order of artists (authors) and their videos (texts).

Considering that the coherence of a compilation is based on the specific order of the clips, as well as on the relationship between the clips and their accompanying titles and other non-authorial interventions throughout the video – Wright’s system of classification de-facto obscures the structure and contents of a compilation [60–62]. And still, the catalog contains a wealth of information about non-authorial literary practices found in scribal notes, tables of contents, indices, rubrications, etc.

So, how could a non-author-oriented study benefit from the richness of the BL catalog, even though the catalog itself hinders such a perspective?

Acknowledging this situation, the limitations of the catalog along with the significance of the data it provides, Michelson and his team began to convert the entire catalog into a digitized database.

In Michelson’s words:

By employing such digital tools [i.e., the digitization of the catalog], Syriaca.org aims to allow the histories of Syriac literature to be told in a way that better preserves the multiple perspectives and diverse theological and literary traditions that produced these manuscripts.[60]

The digitization of Wright’s catalog is accomplished by consistently assigning a specific tag to each identifiable piece of information in Wright’s description of a manuscript, including the textual contents, scribal notes, tables of contents, indices, etc., resulting in a digitized database representing the entire catalog. In total, the database consists of 21,734 different items, which can be filtered and analyzed according to any specific feature (see: https://github.com/srophe/britishLibrary-data.git). For example, one could create a dataset of all the scribal notes mentioned throughout the catalog, and to quantitatively analyze their diachronic and genre distributions, their overall length, the most frequent words, etc. Yet, as Wright’s classification of non-authorial practices is mostly anecdotal, a quantitative study of these practices that relies on precise and accurate data is somewhat problematic. Nevertheless, as seen above in Wright’s description of BL 12,155 (e.g., Basil, in yellow, and one group of excerpts, in red), Wright paid very close attention to excerpts of authors cited in each manuscript, so that the tagging of excerpts is quite reliable. Consequently, filtering out all items that do not represent excerpts, one could produce a dataset of all the excerpts, a total of 19,961, from 998 datable BL mss., with information about genre and date as provided by Wright. In other words, the data about the citations of texts of authors found in the BL mss., as classified by Wright, could be re-oriented as data about excerpts of manuscripts, regardless of their literary taxonomy. Now, we must decide what quantitative feature of excerpts can be analyzed in a meaningful manner.

### Feature selection

How would one analyze the created dataset in a way that will reveal something about the practices of excerpting in the BL mss., and especially, without relying primarily on literary taxonomy nor on preconceived periodizations? Similarly, how would one analyze YouTube compilations according to their video-clips, without referring to the genres, the artists, the original videos of the video-clips, or the date of publication? One would have to rely on other aspects of the excerpted units, such as their size and placement within the compilation.

As the length and placement of excerpts cannot be accurately assessed based on the catalog, this study, which wishes to remain on the firmest of grounds, will rely on the number of excerpts per manuscript as the basic quantitative unit of analysis – similar to the number of tiles in a mosaic, or the number of video-clips in a given compilation. As each excerpt represents a single act of selection and placement performed by the non-authorial agent, the number of excepts per manuscript is a rudimentary indication of the non-authorial textual selections per manuscript. Considering the complexity of pre-print manuscripts, and especially MTMs, a simple analysis of the number of textual selections is far from revealing about the nature of the selections, let alone their combined meaning as a literary compilation. Nevertheless, the number of excerpts per manuscript is a metric that expresses a basic feature of the literary practices of compiling-making, corresponding to a material and cultural practice of text selection and placement.

And so, the data about the citations of authors in manuscripts was re-oriented as data about the excerpts in mss., and from this re-oriented data, a new quantitative feature could be computed for each manuscript – the number of excerpts-per-manuscript (EPM). It is this feature, the EPM, that will be used here as the meaningful quantitative unit of analysis. Now, let’s attempt to redescribe the BL Syriac mss. according to their EPMs (the full EPM dataset can be found in: https://github.com/maeirnoam/syriac-excerpting-practices.git).

## Analysis

As the quantification of Syriac material philology, and particularly of Syriac excerpting, is at its early stages, the venture into the quantitative realm must be very cautious. Now that the quantitative feature of the Syriac mss. was defined, EPM, before making any more assumptions, one should ask – what can be said about the Syriac mss. of the BL when only looking at the distribution of the excerpts-per-manuscript (EPM)? How would one describe the mss. based only on this feature?

From the 998 datable Syriac mss. of the BL collection, regardless of genre or date, 75% of the mss. have 20 excerpts or less (436 (44%), have 1-5 extracts, 158 (16%) 6-10 excerpts, 154 (15%) 11-20 excerpts), and another 164 (17%) have 21-50 excerpts. After them, 57 (5%) mss. have 51-100 excerpts, 27 (2%) mss. have 101-500 excerpts, and 2 mss. have more than 500 excerpts, 660 (BL Add. 14,532) and 1190 (BL Add. 12,155) (see Table 1 and Chart 1). That is, EPM in the BL manuscripts appears to have a non-normal distribution, with most of the manuscripts (748) having low EPMs (1-20), and very few mss. (29) having much higher EPMs (100-1,190).

**Table 1:** Distribution of mss. by EPM range in the Syriac collection of the British Library.

EPM Range	Number of Manuscripts
1-20	748
21-50	164
51-100	57
101-1190	29

The newly computed quantitative feature, EPM, enables to redescribe mss. in a manner that is unrelated to other classifications, literary or temporal. The redescription, the quantitative charting, reveals, simply, that there is a large group of mss. characterized by low EPMs, and a very small group of mss. that share a much higher EPM.

What happens when, in addition to EPM, the genre classification, the original classification by Wright, is allowed within the quantitative chart? How does the EPM of mss. behave within the genre framework? Rather than using a single metric, such as the average EPM, in order to get a more nuanced picture, the minimum and maximum EPM values will be charted, so that the EPM range within each genre will be expressed. Each of the following charts corresponds to a specific genre of mss., such as “Biblical Manuscripts”, “Lives of Saints” or “History”, and within each chart one can find a representation of the EPM ranges of the “Sub-Sections” within that genre.

**Fig 3:**
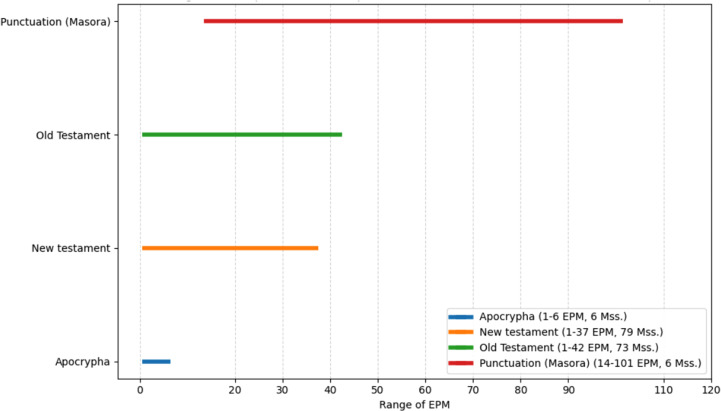
Range of Excerpts Per Manuscript (EPM) in the Sub-Genres of Biblical Manuscripts.

**Fig 4:**
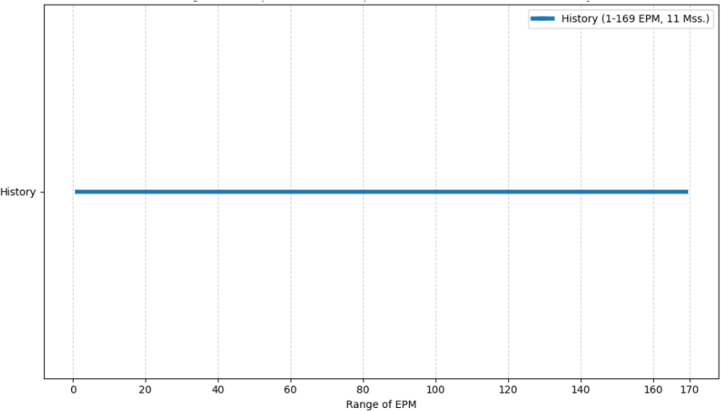
Range of Excerpts Per Manuscript (EPM) in the Sub-Genre of History.

**Fig 5:**
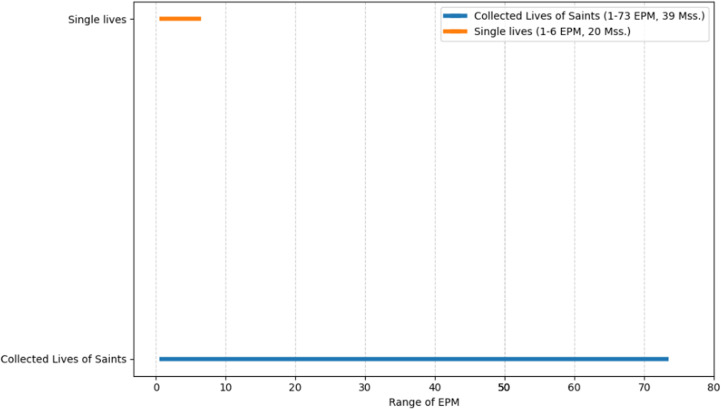
Range of Excerpts Per Manuscript (EPM) in the Sub-Genres of Lives of Saints.

**Fig 6:**
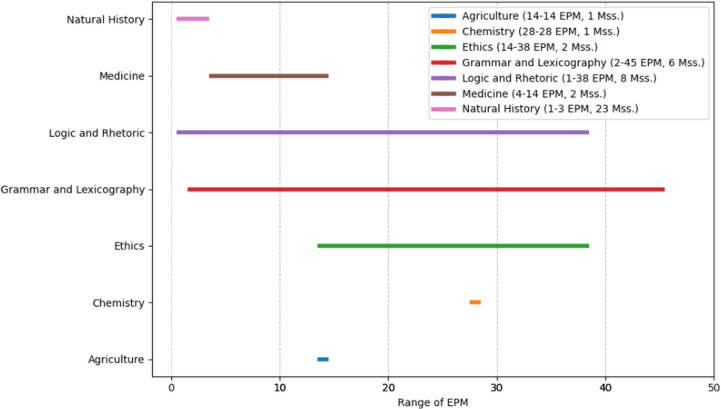
Range of Excerpts Per Manuscript (EPM) in the Sub-Genres of Scientific Literature.

**Fig 7:**
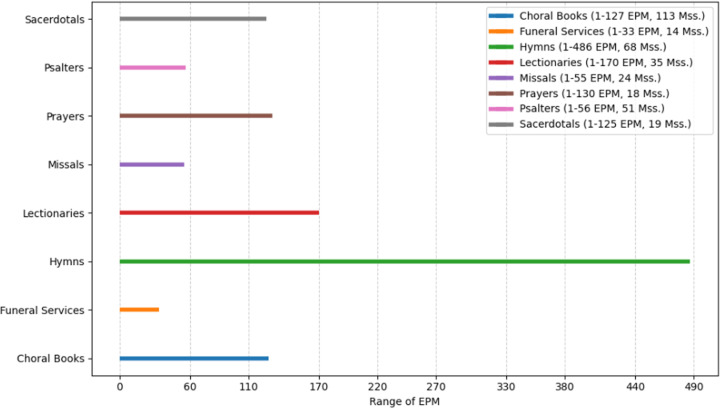
Range of Excerpts Per Manuscript (EPM) in the Sub-Genres of Service Books.

**Fig 8:**
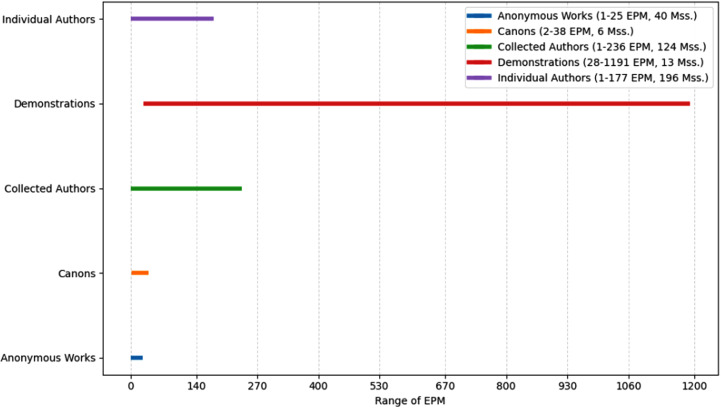
Range of Excerpts Per Manuscript (EPM) in the Sub-Genres of Theology.

As seen in the figures above (Figs 3–8), the mss. grouped by the genres of Wright’s classification, express varying EPM ranges. Biblical manuscripts range from 1-101 EPM, (mostly 1-50, and two additional mss. with 56 and 101 EPM – Add. 12,138 and Add. 12,178, from the sub-section of Punctuation (Masora)). History mss. range from 1-169, (mostly from 1-20, and two additional mss. with 24 and 169 EPM – Add. 25,875 and Add. 17,202). Service Books (i.e., liturgy) range from 1-486 EPM. Scientific Literature range from 1-45 EPM. Lives of Saints ranges from 1-73, including Collected Lives. In Theology, sub-genres include: Individual Authors ranges from 1-177 EPM, (mostly 1-100, and two additional mss. with 147 and 177 EPM – Add. 12,159 and Add. 12,157). Collected authors ranges from 1-236 EPM. Catenae Patrum and Demonstrations against Heresies (abbreviated as Demonstrations) ranges from 28-1190 EPM, mostly 91 + , with Add. 17,195 having 28 EPM. Anonymous Works and Councils of the Church and Ecclesiastical Canons (abbreviated as Canons) span from 1-50 (25 and 38) EPM.

In other words, as seen in the charts above, mss. found in some of Wright’s genres span across a wide EPM range (demonstrations, service books, history, collected and individual authors), while others have a more limited range (biblical, scientific literature, and lives of saints). Moreover, evidently, the small group of mss. with high EPMs that was identified above, is found in several genres, and the large group of mss. with low EPMs is found across all genres. Before considering some possible interpretations of these results, let’s examine the quantitative charting of the mss. using EPM as well as the date, to add an additional diachronic lens.

The BL mss. were divided into 4 groups, according to their EPM: 1%-75% (1-20 EPM); 76%-92% (21-50 EPM); 93%-98% (51-100 EPM); 99%-100% (101-1,190 EPM). What is the diachronic map of each of these groups? What patterns are visible? As with the genre analysis, the minimum and maximum values will be charted, so that the EPM range will be expressed. As mentioned, distant reading does not necessitate an a-priori periodization of the data. As such, we may view the data with the widest temporal lens and allow the periodization to emerge as a result.

**Fig 9:**
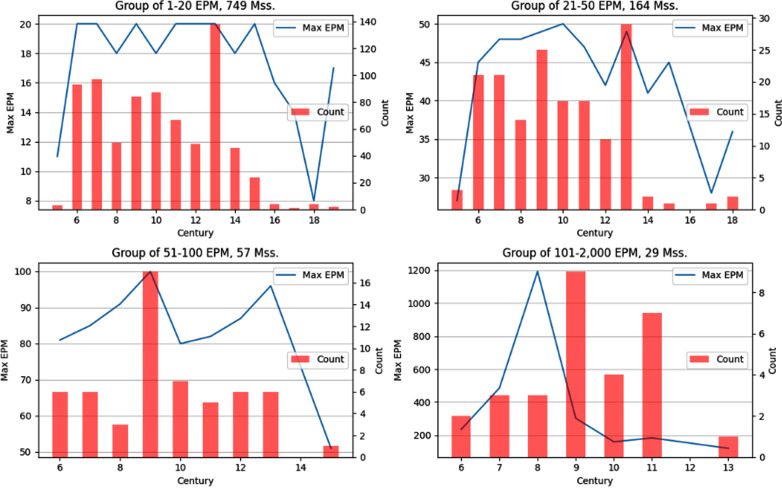
Diachronic behavior of EPM groups over centuries.

As seen in the emerging diachronic charts of Fig 9, both of the lower EPM groups (1-50 EPM, 92% of mss.) span from the 5^th^ until at least the 18^th^ centuries CE, while the 3^rd^ group (51-100 EPM, 5% of mss.) spans from the 6^th^-15^th^ centuries CE, and the 4^th^ group (101-1,190 EPM, 2% of mss.) spans from the 6^th^-13^th^ centuries CE. In addition, within the 4^th^ group, mss. with values higher than 200 EPM are found during a limited period, from the 6^th^-9^th^ centuries CE. Thus, mss. with low EPMs are found throughout the history of Syriac manuscript culture, while mss. with high EPMs are found in a more limited timeframe. Now, let us consider our results.

## Discussion

In this study, a quantitative feature of mss., the EPM, was produced from the digitized BL catalog as a measure of the non-authorial literary practice of excerpting, in an attempt to offer a “big picture” charting of this practice. Three charts were produced, based on simple analyses of descriptive statistics of the quantitative, literary and diachronic distributions of EPMs in the Syriac mss. of the British Library. As mentioned, the quantification of Syriac material philology is in its early stages, therefore, employing quantitative arguments about excerpting needs to be extremely cautious. I would like to make, with much caution, three simple observations regarding the three maps.

First, a simple quantitative distribution chart produced the most basic redescription of the BL mss. according to their EPMs. The chart reveals that most mss. (91%) have low-mid EPMs (1-50 EPM), with nearly half of the mss. (44%) presenting very low EPMs (1-5 EPM), while few (29, i.e. 2%) have much higher (100-1,190 EPM). The selection and arrangement of a large number of excerpts, or textual units (100+), together with the performance of other kinds of non-authorial practices, all intertwined within a single manuscript, would constitute an immense intellectual and literary achievement. As such, considering the complex nature of the literary collection and the network of literary practices (intra- and inter-manuscript) implicated in its production, the rarity of mss. with high EPMs is to be expected [17,23]. And still, it should not be lost upon us that when charting the territory of the BL mss. according to EPM, the resulting topography outlines a small area of higher ground, towering over the land (see Table 2 below).

**Table 2:** The 29 manuscripts with the highest EPM values.

Shelfmark	Wright Section	Wright Sub-Section	EPM	Date
Add. 12,155	Theology	Demonstrations	1191	8
Add. 14,532	Theology	Demonstrations	678	10
Add. 17,143	Service Books	Hymns	487	7
Add. 12,154	Theology	Demonstrations	303	6
Add. 14,533	Theology	Demonstrations	277	9
Add. 12,156	Theology	Collected Authors	236	6
Add. 17,193	Theology	Demonstrations	195	9
Add. 12,144	Theology	Individual Authors	184	11
Add. 12,157	Theology	Individual Authors	177	10
Add. 17,923	Service Books	Lectionaries	170	11
Add. 17,202	History	History	169	6
Add. 17,194	Theology	Demonstrations	168	9
Add. 14,488	Service Books	Lectionaries	160	10
Add. 14,538	Theology	Demonstrations	160	10
Add. 12,159	Theology	Individual Authors	147	9
Add. 12,168	Theology	Demonstrations	143	9
Add. 14,485	Service Books	Lectionaries	133	9
Add. 14,517	Service Books	Prayers	130	11
Add. 14,503	Service Books	Choral Books	127	11
Add. 14,493	Service Books	Sacerdotals	125	10
Add. 17,191	Theology	Demonstrations	124	8
Add. 18,714	Service Books	Lectionaries	121	13
Add. 14,535	Theology	Collected Authors	120	9
Add. 12,146	Service Books	Choral Books	116	11
Add. 12,165	Theology	Collected Authors	113	11
Add. 14,514	Service Books	Hymns	107	7
Add. 16,612	Theology	Collected Authors	107	7
Add. 17,214	Theology	Demonstrations	104	7
Add. 12,178	Biblical Manuscripts	Punctuation (Masora)	101	8

Second, when adding the literary (genre) lens to our map, the quantitative distribution is divided according to the genres of the original BL catalog, revealing EPM patterns within genres, and enabling a cross-genre comparison. Interestingly, the EPMs of mss. in all genres exhibit a measure of variability seen in various ranges of EPMs, with the smallest range (1-38 EPM) found in biblical mss. and the largest (51-1190) in demonstrations. One could argue that at least for some of the genres, a genre’s EPM range corresponds to an inherent logic and structure of the genre. For instance, one could claim that the low EPM range of biblical mss. reflects the authoritative status of biblical texts, while the extremely large EPM range of demonstrations reflects the innovative and argumentative nature of this genre. Yet, the sample size of the data from the genres is arguably insufficiently large – e.g., there are only 13 mss. in the demonstrations genre – and the genre classification itself is based on 19^th^ century logic that is somewhat outdated, so that claims about the structural characterization of genre should be treated as conjectures rather than solid arguments.

While EPM ranges of the mss. of the BL genres can be used to attempt to describe differences between genres, they can also reveal similarities, assuming we accept provisionally the genre classification. Here one notices that all genres include mss. with low-mid EPMs (1-50 EPM), and that several genres – demonstrations, collected authors (theology), individual theology, history and liturgy – each have mss. with high EPMs (over 100 EPM). That is, low-mid excerpting is a literary practice that is found in *all* genres, and high excerpting is found in much less genres. Moreover, the genres with high excerpting include genres that are associated with literary collections, such as demonstrations, collected authors (theology) and history, as well as genres that are not, such as individual theology and liturgy. In other words, data about excerpting, low-mid-high, is not contained in any one genre, but is found across multiple genres. And still, the genre distribution of high excerpting is much more limited compared to that of low-mid excerpting, so that the topographical distinction between the higher and lower grounds is still maintained.

Third, adding a diachronic framework enables to chart the gradual accumulation of the quantitative distribution. The resulting diachronic charts reveal that low-mid EPMs continue to be found at least until the 18^th^ century, while high-EPMs (100 + EPM) are limited to mss. of the 6^th^-13^th^ centuries, with the highest values (200+) found only during the 6^th^-9^th^ centuries. One could argue that excerpting patterns, measured via EPM, could serve to characterize historical periods, as was the case for genre analysis. For instance, we have seen that excerpting is considered an important, if not essential, cultural feature of Christianity in late antiquity. Indeed, the 6^th^-9^th^ centuries CE, considered by some to be a part of late antiquity, were characterized by the highest EPM values, and so perhaps this can serve as proof of the prominence of excerpting in late antiquity. Yet, as was true for the genre analysis, one must treat the historical claims as mere conjectures, due to the limited sample size and the dating of many of the mss., which are based on Wright’s 19^th^ century estimations. One would expect that including Syriac mss. from other collections and reexamining the dating of the BL mss. via current codicological and paleographical methods will alter the results significantly. In any case, the diachronic distribution of high excerpting is much shorter, when compared to that of low-mid excerpting, so that the topographical distinction between the higher and lower grounds is still maintained.

Based on the emerging topography of Syriac excerpting, I would like to offer interpretations that may be relevant to the material-philology-oriented study of non-authorial literary practices and excerpting in particular. First, as seen, data about EPMs, the quantitative feature of excerpting, is found across genres and time, i.e., the traditional literary and historical classifications of the BL mss. do not neatly contain the quantitative behavior of EPM. This seems to suggest that studies grounded in material philology perspectives, which are vested in the proper study of non-authorial practices, might find it useful to seriously consider applying cross-genre and diachronic analyses to their data, as well as furthering attempts to develop material-philology-oriented classifications.

Second, EPM, the quantitative feature of the non-authorial practice of excerpting that was produced here, serves as a category of analysis that is unhindered by the traditional classification. This facilitated the loss of the genre- and temporal-oriented categories and the redescription of the BL mss. according to the newly produced quantitative feature. In doing so, a new topography of Syriac mss. emerged, in which a small group of mss. with high EPM values stands out, as a mountain peak towering over the land, that may serve as an anchor to navigate through the uncharted territories of Syriac non-authorial literary practices. As mentioned, scholars of Syriac literary collections would not be surprised by the small number of mss. that are part of the highest terrain, as manuscripts with high EPM values would represent immense intellectual and literary achievements. Taking into account that excerpts, defined simply as textual selections or textual units, are usually linked to other intra- and inter-manuscript units, one can argue that mss. with high EPMs could be considered rich repositories of non-authorial literary practices. As such, the mss. that represent the higher terrain may serve as major sites or intersections of Syriac literary culture, that warrant further close studies.

In conclusion, this paper set out to contribute to the emerging field of material philology and the study of non-authorial literary practices, by offering a “big picture” analysis, a distant reading, of Syriac excerpting. The distant reading enabled to examine the utility of the traditional classification in describing quantitative features of excerpting, suggesting that it is insufficient, and that furthering a material-philology-oriented classification is warranted. The digitization of the BL’s Syriac manuscripts, still a desideratum, could offer a significant way forward, facilitating such a renewed classification as well as an expansion of the EPM data. Lastly, the distant reading not only enabled to complement the traditional classification, but also to reclassify the BL Syriac mss., via the EPM measurement, in a way that is more aligned with material philology, thereby establishing relationships and inviting comparisons between mss. that were previously unrelated. Accordingly, a small group of mss. with high EPMs, representing rich repositories of non-authorial practices, emerged as pinnacles of Syriac literary culture. As such, future research might benefit from conducting cross-genre comparisons of high-EPM manuscripts, including both theological and non-theological genres. As studies, especially close readings, continue, the significance of these manuscripts will become clearer.
